# CIDEC/FSP27 exacerbates obesity-related abdominal aortic aneurysm by promoting perivascular adipose tissue inflammation

**DOI:** 10.1093/lifemeta/loae035

**Published:** 2024-09-18

**Authors:** Qing Zhu, Da Luo, Yining Li, Liyang Yu, Zixuan Zhang, Feng Ouyang, Liangkui Li, Manxi Lu, Changyong Hu, Yinuo Dong, Chengxin Ma, Yan Liang, Tong-Jin Zhao, Feng-Jung Chen, Peng Li, Tian-Shu Yang

**Affiliations:** Shanghai Key Laboratory of Metabolic Remodeling and Health, Institute of Metabolism and Integrative Biology, Institutes of Biomedical Sciences, Fudan University, Shanghai 200438, China; Shanghai Key Laboratory of Metabolic Remodeling and Health, Institute of Metabolism and Integrative Biology, Institutes of Biomedical Sciences, Fudan University, Shanghai 200438, China; Shanghai Qi Zhi Institute, Shanghai 200030, China; Shanghai Key Laboratory of Metabolic Remodeling and Health, Institute of Metabolism and Integrative Biology, Institutes of Biomedical Sciences, Fudan University, Shanghai 200438, China; State Key Laboratory of Membrane Biology and Tsinghua-Peking Center for Life Sciences, Beijing Advanced Innovation Center for Structural Biology, School of Life Sciences, Tsinghua University, Beijing 100086, China; Shanghai Key Laboratory of Metabolic Remodeling and Health, Institute of Metabolism and Integrative Biology, Institutes of Biomedical Sciences, Fudan University, Shanghai 200438, China; Shanghai Key Laboratory of Metabolic Remodeling and Health, Institute of Metabolism and Integrative Biology, Institutes of Biomedical Sciences, Fudan University, Shanghai 200438, China; State Key Laboratory of Membrane Biology and Tsinghua-Peking Center for Life Sciences, Beijing Advanced Innovation Center for Structural Biology, School of Life Sciences, Tsinghua University, Beijing 100086, China; Shanghai Key Laboratory of Metabolic Remodeling and Health, Institute of Metabolism and Integrative Biology, Institutes of Biomedical Sciences, Fudan University, Shanghai 200438, China; Shanghai Key Laboratory of Metabolic Remodeling and Health, Institute of Metabolism and Integrative Biology, Institutes of Biomedical Sciences, Fudan University, Shanghai 200438, China; Shanghai Key Laboratory of Metabolic Remodeling and Health, Institute of Metabolism and Integrative Biology, Institutes of Biomedical Sciences, Fudan University, Shanghai 200438, China; Shanghai Key Laboratory of Metabolic Remodeling and Health, Institute of Metabolism and Integrative Biology, Institutes of Biomedical Sciences, Fudan University, Shanghai 200438, China; Shanghai Key Laboratory of Metabolic Remodeling and Health, Institute of Metabolism and Integrative Biology, Institutes of Biomedical Sciences, Fudan University, Shanghai 200438, China; Shanghai Key Laboratory of Metabolic Remodeling and Health, Institute of Metabolism and Integrative Biology, Institutes of Biomedical Sciences, Fudan University, Shanghai 200438, China; Shanghai Key Laboratory of Metabolic Remodeling and Health, Institute of Metabolism and Integrative Biology, Institutes of Biomedical Sciences, Fudan University, Shanghai 200438, China; Shanghai Qi Zhi Institute, Shanghai 200030, China; Shanghai Key Laboratory of Metabolic Remodeling and Health, Institute of Metabolism and Integrative Biology, Institutes of Biomedical Sciences, Fudan University, Shanghai 200438, China; Shanghai Qi Zhi Institute, Shanghai 200030, China; State Key Laboratory of Membrane Biology and Tsinghua-Peking Center for Life Sciences, Beijing Advanced Innovation Center for Structural Biology, School of Life Sciences, Tsinghua University, Beijing 100086, China; School of Life Sciences, Tianjian Laboratory of Advanced Biomedical Sciences, Zhengzhou University, Zhengzhou, Henan 450001, China; Shanghai Key Laboratory of Metabolic Remodeling and Health, Institute of Metabolism and Integrative Biology, Institutes of Biomedical Sciences, Fudan University, Shanghai 200438, China; Shanghai Qi Zhi Institute, Shanghai 200030, China; Shanghai Key Laboratory of Lung Inflammation and Injury, Department of Pulmonary Medicine, Zhongshan Hospital, Fudan University, Shanghai 200032, China

**Keywords:** AAA, inflammation, PVAT, *Cidec*, CCL2

## Abstract

Abdominal aortic aneurysm (AAA) is strongly correlated with obesity, partially due to the abnormal expansion of abdominal perivascular adipose tissue (PVAT). Cell death-inducing DNA fragmentation factor-like effector C (CIDEC), also known as fat-specific protein 27 (FSP27) in rodents, is specifically expressed in adipose tissue where it mediates lipid droplet fusion and adipose tissue expansion. Whether and how CIDEC/FSP27 plays a role in AAA pathology remains elusive. Here, we show that FSP27 exacerbates obesity and angiotensin Ⅱ (Ang Ⅱ)-induced AAA progression. FSP27 deficiency in mice inhibited high-fat diet-induced PVAT expansion and inflammation. Both global and adipose tissue-specific FSP27 ablation significantly decreased obesity-related AAA incidence. Deficiency of FSP27 in adipocytes abrogated matrix metalloproteinase-12 (MMP12) expression in aortic tissues. Infiltrated macrophages, which partially colocalize with MMP12, were significantly decreased in the FSP27-deficient aorta. Mechanistically, knockdown of *Fsp27* in 3T3-L1 adipocytes inhibited C–C motif chemokine ligand 2 (CCL2) expression and secretion through a c-Jun N-terminal kinase (JNK)-dependent pathway, thereby leading to reduced induction of macrophage migration, while *Cidec* overexpression rescued this effect. Overall, our study demonstrates that CIDEC/FSP27 in adipose tissue contributes to obesity-related AAA formation, at least in part, by enhancing PVAT inflammation and macrophage infiltration, thus shedding light on its significance as a key regulator in the context of obesity-related AAA.

## Introduction

Abdominal aortic aneurysm (AAA) is a degenerative vascular disease characterized by progressive structural deterioration and dilation of the aortic wall [1, 2]. AAA mostly affects aged men and accounts for 150,000−200,000 deaths globally every year [3]. Most patients with AAA are usually asymptomatic until the aneurysm ruptures, resulting in a mortality rate of 85%−90% [4, 5]. While progress has been made in understanding the underlying mechanisms of AAA, there is no effective therapy at present to prevent AAA progression, emphasizing the need to better understand AAA molecular pathophysiology.

Obesity is an important risk factor for AAA [6–12]. A previous study has shown that people with high body mass index (BMI) are more likely to develop AAA than those with lower BMI [13]. In addition to their key roles in energy storage and expenditure, adipose tissues secrete bioactive molecules in response to physiological stimulation and metabolic stress, with some of these secretory factors regulating cardiovascular remodeling [14]. Perivascular adipose tissue (PVAT), a unique type of adipose tissue that surrounds the big aortic vessels, is thought to play a role in obesity-induced AAA. Except for providing mechanical support for aorta, PVAT is also a crucial endocrine tissue that modulates vascular function [15]. Under normal physiological conditions, PVAT exerts an anticontractile effect on the vasculature by releasing vasoactive molecules. In the context of obesity, PVAT expands dramatically and becomes dysfunctional, leading to pro-inflammatory cytokine release [15–19] and immune cell infiltration in aortic aneurysms [20–22], which may contribute to AAA formation. In AAA, macrophages are the most abundant immune cells that reside in the aortic wall and contribute to AAA progression by producing proteolytic enzymes, specifically matrix metalloproteinases (MMPs) and cytokines [16]. Although previous study has suggested the correlation between obesity-induced PVAT inflammation and AAA formation, direct evidence supporting the casual relationship between PVAT inflammation and AAA formation in the context of obesity is still lacking [17].

Cell death-inducing DNA fragmentation factor-like effector C (CIDEC), also known as fat-specific protein 27 (FSP27) in mice, is a lipid droplet (LD)-associated protein that plays an important role in lipid storage in adipose tissue [18, 19]. Studies from our group and others reported that CIDEC/FSP27 promotes LD growth by mediating the exchange and transfer of lipid at the contact sites of LDs [20–23]. A recent study from our group demonstrated that mice lacking *Fsp27* are protected from high-fat diet (HFD) or leptin deficiency (*ob/ob*)-induced obesity due to lipodystrophy [20]. However, the role of CIDEC/FSP27 in PVAT dysfunction and AAA pathogenesis remains unknown.

Given that CIDEC/FSP27 is implicated in lipid storage and adipose tissue expansion, we hypothesize that it may play a role in the development of obesity-related AAA. In the present study, we demonstrated that CIDEC/FSP27 exacerbates PVAT inflammation in HFD-induced obesity and consequently promotes HFD and angiotensin Ⅱ (Ang Ⅱ)-induced AAA progression. Our findings expand our knowledge of the role of PVAT in AAA and highlight the potential of CIDEC/FSP27 as a therapeutic target for obesity-related AAA.

## Results

### Reduced fat mass and inflammation in PVAT of *Fsp27*^−/−^ mice

**Figure 1:**
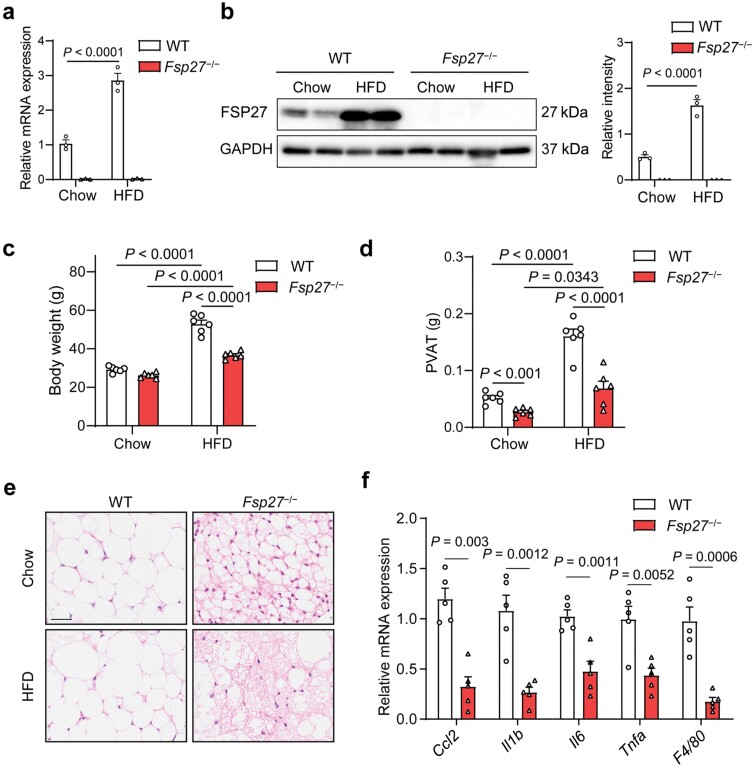
PVAT fat mass and inflammation are reduced in *Fsp27*^−/−^ mice. (a and b) Relative mRNA (a) and protein (b) levels of FSP27 in PVAT of WT and *Fsp27*^−/−^ mice fed with a chow diet or an HFD for three months were determined by qPCR (a) and western blot analysis (b). (c and d) Body weight (c) or weight of abdominal adipose tissues (d) from WT and *Fsp27*^−/−^ mice fed with a chow diet or an HFD for three months (*n* = 6). (e) Hematoxylin and eosin (H&E) staining of abdominal adipose tissues dissected from WT and *Fsp27*^−/−^mice fed with a chow diet or an HFD for three months. Scale bar, 50 μm. (f) Relative mRNA expression levels of inflammatory genes in PVAT from WT and *Fsp27*^−/−^ mice fed with an HFD for three months (*n* = 5). Data are presented as the means ± SEM. *P* values were calculated by two-way ANOVA with Bonferroni test (a, c, and d) and two-tailed unpaired Student’s *t*-test (f).

To determine whether CIDEC/FSP27 contributes to HFD-induced PVAT expansion and inflammation, we first compared the expression level of *Fsp27* in abdominal PVAT in wild-type (WT) and *Fsp27* knockout (*Fsp27*^**−/−**^) mice fed with chow or a 60% HFD for three months. The results showed that *Fsp27* mRNA and protein levels in abdominal PVAT were significantly upregulated in response to HFD treatment (Fig. 1a and b). As previously reported, *Fsp27*^**−/−**^ mice weighed about 40% less than the controls after 12-week HFD treatment (Fig. 1c), and the volume of both gonadal and subcutaneous fat was significantly reduced in *Fsp27*^**−/−**^ mice (Supplementary Fig. S1a and b). In mice, it is recognized that PVAT around the abdominal and thoracic aortas have the features of white adipose tissue (WAT) and brown adipose tissue (BAT), respectively. We observed that the weight of abdominal PVAT was significantly decreased in *Fsp27*^**−/−**^ mice fed with a chow diet or an HFD, whereas no difference was observed in the weight of thoracic PVAT between *Fsp27*^**−/−**^ mice and WT mice (Fig. 1d; Supplementary Fig. S1c). Given that the abdominal PVAT is considered to contribute to AAA progression, we focus on abdominal PVAT, which is hereinafter referred to as PVAT. Histological analysis of PVAT from WT and *Fsp27*^**−/−**^ mice showed that perivascular adipocytes from *Fsp27*^**−/−**^ mice were characterized by multilocular LDs when fed with a chow or an HFD (Fig. 1e). We next investigated whether PVAT from *Fsp27*^**−/−**^ mice is protected from HFD-induced inflammation by analyzing the expression levels of inflammatory genes in PVAT by quantitative real-time polymerase chain reaction (qPCR). We observed significantly reduced expression levels of *Ccl2* (C-C motif chemokine ligand 2)*, Tnfa* (tumor necrosis factor-α), *Il6* (interleukin-6), and *Il1b* (interleukin-1β), as well as *Adgre1* (F4/80, a macrophage-specific marker) in the PVAT of *Fsp27*^**−/−**^ mice (Fig. 1f). Overall, FSP27 deficiency leads to resistance against the pathological expansion and inflammation of PVAT induced by HFD.

### FSP27 deficiency attenuates HFD and Ang Ⅱ-induced AAA formation

**Figure 2:**
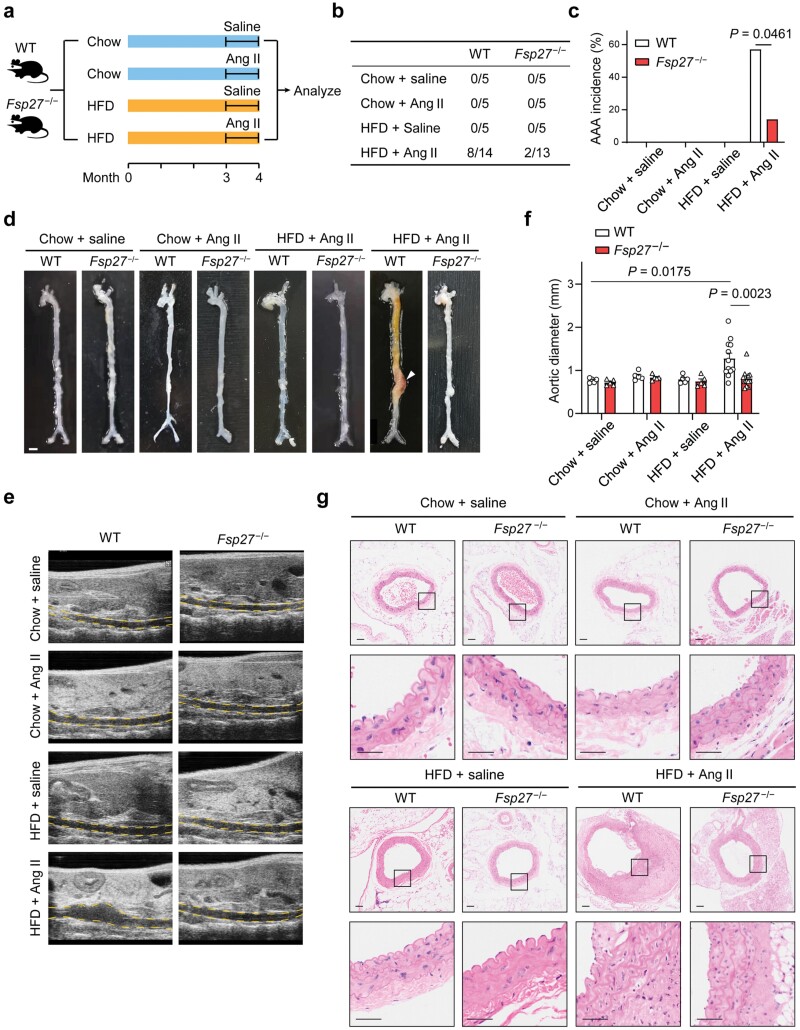
FSP27 deficiency reduces the incidence of HFD and Ang Ⅱ-induced AAA. (a) Schematic experimental design. WT and *Fsp27*^−/−^ mice fed with a chow diet or an HFD for three months were administered with saline or Ang Ⅱ for four weeks to induce AAA. (b) Table showing the incidence of AAA in WT and *Fsp27*^−/−^ mice. (c) The incidence of AAA as described in (a). (d) Representative images showing the features of Ang Ⅱ-induced aneurysms from mice treated as described in (a). The arrow indicates an aorta aneurysm. Scale bar, 2 mm. (e) Representative ultrasound images of the abdominal aortas from mice treated as described in (a). Aorta lumina are indicated by the yellow lines. (f) The quantitative results of (e). (g) Representative H&E staining of the abdominal aortas from mice treated as described in (a). Scale bar, 50 μm. Data are presented as the means ± SEM in (e). *P* values were calculated by Fisher’s exact test (c) and two-way ANOVA with Bonferroni test (f).

We next investigated the role of CIDEC/FSP27 in the development of AAA triggered by HFD-induced obesity. The current widely used murine AAA models include Ang Ⅱ infusion in apolipoprotein E deficient (*ApoE*^**−/−**^) mice, elastase infusion, and calcium chloride exposure, which are not physiologically relevant to obesity-related AAA in humans. To better study the involvement of PVAT in obesity-related AAA *in vivo*, we induced AAA development by using mice fed with an HFD and infused with Ang Ⅱ. Both WT and *Fsp27*^**−/−**^ mice were fed with HFD for three months and then subjected to Ang Ⅱ infusion or sham treatment for four weeks (Fig. 2a). In total, HFD and Ang Ⅱ infusion led to the development of AAA in 57% (8 of 14) of WT mice, including 14% (2 of 14) mortality from aneurysm rupture. In contrast, only 15% (2 of 13) of *Fsp27*^**−/−**^ mice developed AAA, with 7% (1 of 14) mortality from aneurysm rupture (Fig. 2b and c). Either HFD or Ang Ⅱ treatment only did not induce AAA formation or death. Morphologically, aorta expansion in *Fsp27*^**−/−**^ mice fed with an HFD and infused with Ang Ⅱ was significantly reduced (Fig. 2d). We did not observe any incidence of aneurysms in mice treated with Ang Ⅱ alone. However, it is noteworthy that five-month-old C57BL/6J mice treated with Ang Ⅱ at a dosage of 1 μg/kg/min resulted in an approximate 10%−20% incidence of aneurysms. Our observation may be attributed to a sampling bias, as the group consisted of only five mice, which may not accurately reflect the true incidence of aneurysms. No significant differences in systolic blood pressure were observed between WT and *Fsp27*^**−/−**^ mice (Supplementary Fig. S2). Ultrasonography further confirmed a significant reduction in the diameter of the abdominal aorta in *Fsp27*^**−/−**^ mice (Fig. 2e and f). Histologically, cellular and architectural changes of typical AAA induced by HFD and Ang Ⅱ treatment, including thrombus formation and adventitial remodeling, were significantly decreased in *Fsp27*^**−/−**^ mice compared to WT mice (Fig. 2g). In addition, we observed no obvious structural difference in the formed aneurysms between WT and *Fsp27*^**−/−**^ groups. These results indicated that the main difference between WT and *Fsp27*^**−/−**^ groups lied in the incidence of AAA (Supplementary Fig. S4). Together, these results suggest that Cidec/FSP27 deficiency protects against HFD and Ang Ⅱ-induced AAA formation.

To understand whether FSP27 deficiency affects the development of thoracic aortic aneurysms and dissection (TAAD), we analyzed echocardiography and found that the incidence of TAAD was comparable in WT (1 in 9) and *Fsp27*^**−/−**^ mice (1 in 7) fed with an HFD and infused with Ang Ⅱ (Supplementary Fig. S5a). Although there was an increase in the luminal diameter of the descending aorta in *Fsp27*^**−/−**^ mice, this observed change did not reach statistical significance. It is noteworthy that the thoracic PVAT from WT mice shared a morphological similarity with BAT, as evidenced by the multilocular appearance of the adipocytes (Supplementary Fig. S5b). In contrast, the thoracic PVAT from *Fsp27*^**−/−**^ mice exhibited similarities to WAT, characterized by adipocytes predominantly displaying a unilocular appearance. This observation aligns with findings from our group and others [24, 25], which showed that the size of LD was markedly larger in the BAT of *Fsp27*^**−/−**^ mice than in that of WT mice. This phenomenon can be explained by the predominant expression of FSP27β isoform in BAT, which contains 10 additional amino acids at the N-terminal domain of the conventional FSP27α isoform in WAT. FSP27β was suggested to promote small multilocular LD formation in BAT by inhibiting the homodimerization of CIDEA [25]. The differential regional susceptibility of the aorta to aneurysm in the context of FSP27 deficiency could be attributed to the dual role of FSP27 in modulating PVAT function across the abdominal and thoracic regions.

### FSP27 deficiency in adipose tissue protects mice from HFD and Ang Ⅱ-induced AAA formation

**Figure 3:**
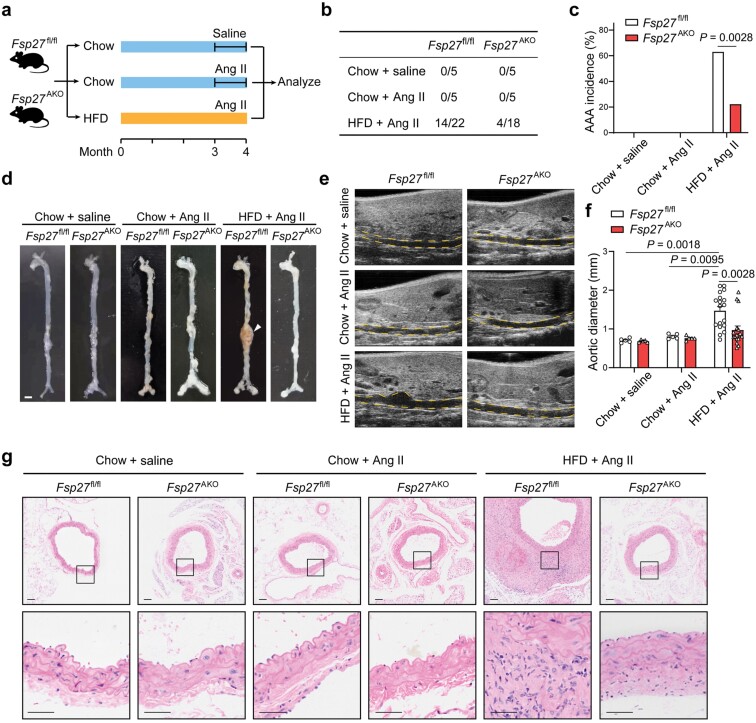
*Fsp27* ablation in adipocytes protects mice from HFD and Ang Ⅱ-induced AAA. (a) Schematic experimental design. In brief, WT and *Fsp27*^−/−^ mice fed with a chow diet or an HFD for three months were administered with Ang Ⅱ for four weeks to induce AAA. (b) Table showing the incidence of AAA in *Fsp27*^fl/fl^ and *Fsp27*^AKO^ mice. (c) Bar graph showing incidence of AAA as described in (a). (d) Representative images showing the features of Ang Ⅱ-induced aneurysms. The arrow indicates an aorta aneurysm. Scale bar, 2 mm. (e) Representative ultrasound images of the abdominal aortas from mice treated as described in (a). Aorta lumina are indicated by the yellow lines. (f) The quantitative results of (e). (g) Representative H&E staining of the abdominal aortas from mice treated as described in (a). Scale bar, 50 μm. Data are presented as the means ± SEM in (f). *P* values were calculated by Fisher’s exact test (c) and two-way ANOVA with Bonferroni test (f).

To further elucidate the contribution of adipocyte-derived CIDEC/FSP27 in AAA formation, we generated adipocyte tissue-specific *Fsp27* knockout mice (*Fsp27*^AKO^) by crossing *Fsp27*^flox/flox^ (*Fsp27*^fl/fl^) mice with Cre transgenic mice expressing the Cre recombinase under the control of adipocyte-specific adiponectin promoter (*Adipoq-*Cre). The efficiency of *Fsp27* knockout in PVAT was verified by qPCR and western blot analysis (Supplementary Fig. S3a and b). Both *Fsp27*^fl/fl^ and *Fsp27*^AKO^ mice were fed with an HFD for three months and infused with saline or Ang Ⅱ for four weeks (Fig. 3a). Adipocyte-specific deletion of *Fsp27* resulted in reduction of body weight and fat mass of PVAT (Supplementary Fig. S3c and d). Consistent with the findings in *Fsp27*^**−/−**^ mice, the incidence of AAA in *Fsp27*^AKO^ mice (22%, 4 of 18, including 1 died from aneurysm rupture) was significantly reduced compared to that of *Fsp27*^fl/fl^ mice (63%, 14 of 22, including 4 died from aneurysm rupture) (Fig. 3b and c). Aortic width expansion induced by HFD and Ang Ⅱ was significantly reduced in *Fsp27*^AKO^ mice compared to *Fsp27*^fl/fl^ mice (Fig. 3d). FSP27 deficiency in adipose tissue significantly reduced HFD and Ang Ⅱ-induced aortic dilation as determined by ultrasound (Fig. 3e and f). We further examined the pathophysiological changes of the aortic tissue. Representative photographs of aortic specimens show that aortic wall thickness was significantly reduced in HFD and Ang Ⅱ-treated *Fsp27*^AKO^ mice compared to *Fsp27*^fl/fl^ mice (Fig. 3g). These data demonstrate that *Fsp27* ablation in adipocyte tissue significantly decreases the incidence of obesity-triggered AAA.

### FSP27 deficiency in adipose tissue reduces MMP12 expression and macrophage infiltration in AAA lesions

**Figure 4:**
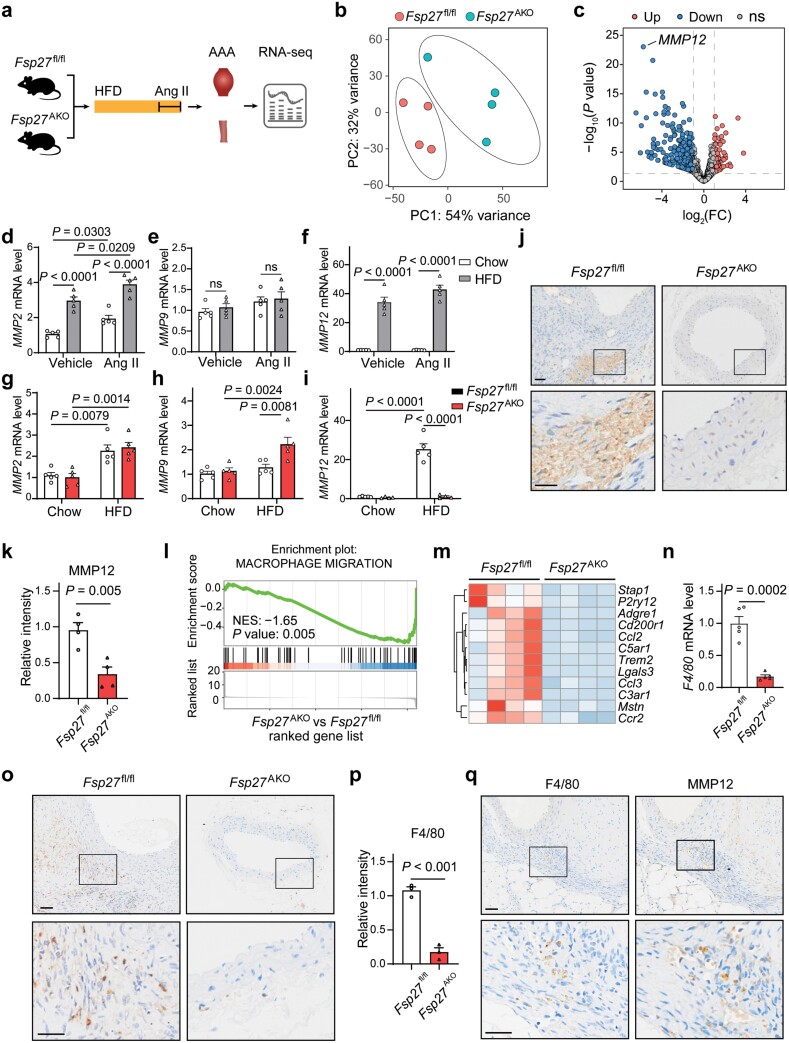
FSP27 deficiency in adipocytes reduces macrophage infiltration in AAA lesions. (a) Schematic illustration of experimental design. *Fsp27*^fl/fl^ and *Fsp27*^AKO^ mice were fed with a chow diet or an HFD for three months and administered with Ang Ⅱ for four weeks. Aortas were subjected to RNA-seq analysis (*n* = 4). (b) PCA score plot of the aortic transcriptome data obtained from mice as described in (a). (c) Volcano plot of DEGs using the same data as in (a) (blue, downregulated; red, upregulated; gray, not significant; adjusted *P* value < 0.05 and absolute fold change (FC) > 2). (d−f) Relative mRNA expression of *Mmp2*, *Mmp9*, and *Mmp12* in the aneurysmal tissues of mice fed with a chow diet or an HFD and then infused with or without Ang Ⅱ (*n* = 5). (g−i) Relative mRNA expression of *Mmp2*, *Mmp9*, and *Mmp12* in the aortas of *Fsp27*^fl/fl^ and *Fsp27*^AKO^ mice fed with a chow diet or an HFD and then infused with Ang Ⅱ (*n *= 5). (j) Immunohistochemical staining of the abdominal aortic sections for MMP12 in *Fsp27*^fl/fl^ and *Fsp27*^AKO^ mice as described in (a). Scale bar, 50 μm. (k) Statistical analysis of MMP12 expression in (j). (l and m) GSEA enrichment plot (l) and heatmap (m) of macrophage migration-associated gene set from the same data as in (a). (n) Relative mRNA expression of *F4/80* in the aneurysmal tissues of *Fsp27*^fl/fl^ and *Fsp27*^AKO^ mice (*n* = 5). (o) Immunohistochemical staining of the abdominal aortic sections for F4/80 in *Fsp27*^fl/fl^ and *Fsp27*^AKO^ mice as described in (a). Scale bars, 50 μm. (p) Statistical analysis of F4/80 expression in (o). (q) Representative immunohistochemical staining in serial sections showing the expression of F4/80 and MMP12 in the aortas of *Fsp27*^fl/fl^ mice treated with HFD and Ang Ⅱ. Data are presented as the means ± SEM. *P* values were calculated by Fisher’s exact test (c), two-way ANOVA with Bonferroni test (d−i), and Student’s *t*-test (m). ns, not significant.

To understand the mechanism by which CIDEC/FSP27 regulates AAA formation, we profiled the transcriptome of aortic samples from both *Fsp27*^fl/fl^ and *Fsp27*^AKO^ mice subjected to an HFD and Ang Ⅱ treatment using RNA sequencing (RNA-seq) (Fig. 4a). Principal component analysis (PCA) of the gene expression profiles revealed a significant separation between *Fsp27*^fl/fl^ and *Fsp27*^AKO^ mice (Fig. 4b). Using adjusted *P* < 0.05 and absolute log_2_(fold change) > 1 as the cutoff criteria, we have identified 883 differentially expressed genes (DEGs), with 113 transcripts upregulated and 770 transcripts downregulated in *Fsp27*^AKO^ mice compared to *Fsp27*^fl/fl^ mice. Notably, one of the most downregulated genes in the aortas of *Fsp27*^AKO^ mice was *Mmp12* (Fig. 4c), which is primarily expressed in macrophages and is involved in the breakdown of extracellular matrix proteins in AAA [26].

The degradation of elastin and collagen in the aneurysm wall by MMPs is recognized as a critical contributor to the pathogenesis of AAA. To assess the involvement of MMPs in obesity-related AAA progression, we examined the expression of MMPs in the aortas of both chow and HFD-fed mice infused with saline or Ang Ⅱ. The results showed that HFD induced a two-fold increase in the expression level of *Mmp2*, whereas there was no significant difference in the expression level of *Mmp9* between the two groups of mice (Fig. 4d and e). The expression of *Mmp13* was barely detectable (data not shown). Notably, *Mmp12* was dramatically increased in mice fed with an HFD compared to those fed with a chow diet, regardless of whether the mice were infused with Ang Ⅱ or not (Fig. 4f), suggesting that MMP12 might be specifically correlated with HFD-induced PVAT inflammation. We next checked the expression levels of MMPs in the aortas from both *Fsp27*^fl/fl^ and *Fsp27*^AKO^ mice fed with either a chow diet or an HFD and infused with Ang Ⅱ. The mRNA levels of *Mmp2* and *Mmp9* were either unchanged or increased in the absence of FSP27, respectively (Fig. 4g and h; Supplementary Fig. S6). In contrast, the mRNA level of *Mmp12* was significantly decreased in the aortas of *Fsp27*^AKO^ mice than those of *Fsp27*^fl/fl^ mice (Fig. 4i; Supplementary Fig. S6). Immunohistochemical analysis further confirmed that MMP12 staining was significantly reduced in the aortas of *Fsp27*^AKO^ mice compared to that of *Fsp27*^fl/fl^ mice (Fig. 4j and k).

Previous studies showed that MMP12 is predominantly secreted by macrophages during AAA formation [27]. Therefore, we investigated the possibility that reduced infiltration of macrophages leads to decreased MMP12 production in the aortas during AAA formation in the absence of FSP27. Gene set enrichment analysis showed that the Gene Ontology (GO) gene sets “macrophage migration” was substantially downregulated in the aortas from *Fsp27*^AKO^ mice (Fig. 4l). The expression levels of most genes associated with these GO gene sets “macrophage migration”, including the chemokine CCL2, were decreased in the aortas of *Fsp27*^AKO^ mice (Fig. 4m). Furthermore, the mRNA level of *F4/80* was significantly reduced in the aortas of *Fsp27*^AKO^ mice (Fig. 4n). Immunohistochemical staining revealed a significant reduction in the number of F4/80^+^ cells in the aortic walls of *Fsp27*^AKO^ mice (Fig. 4o and p). These results indicate a significant decrease in macrophage recruitment in *Fsp27*^AKO^ mice. Furthermore, immunohistochemical staining of MMP12 and F4/80 in serial sections from *Fsp27*^fl/fl^ mice showed a correlation between the positive stained regions of MMP12 and F4/80 (Fig. 4q), indicating that the expression of MMP12 probably derives from macrophages, in accordance with previous studies [27]. Collectively, these findings suggest that MMP12 levels and infiltration of macrophages were diminished in the aortas of *Fsp27*^AKO^ mice in obesity-related AAA.

### FSP27 in adipocytes promotes macrophage recruitment by modulating the production of CCL2

**Figure 5:**
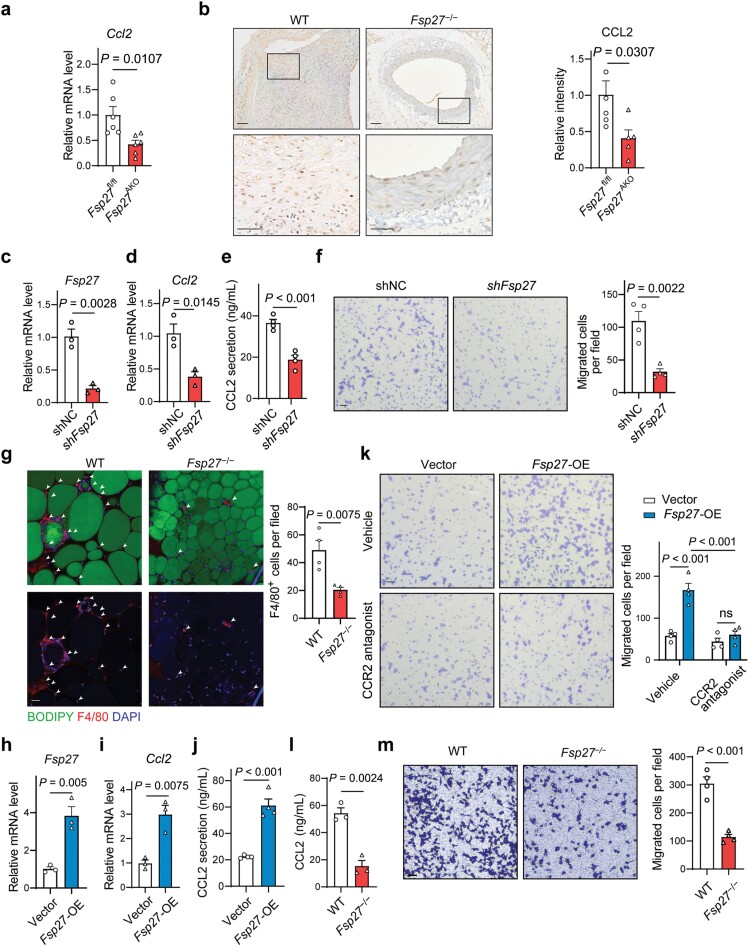
FSP27 in adipocytes promotes macrophage migration through the CCL2/CCR2 axis. (a) Relative mRNA expression of *Ccl2* in the aneurysmal tissues of *Fsp27*^fl/fl^ and *Fsp27*^AKO^ mice fed with an HFD and then infused with Ang Ⅱ (*n* = 6). (b) Immunohistochemical staining of CCL2 and quantitative analysis of positive staining signals in the abdominal aortic sections from *Fsp27*^fl/fl^ and *Fsp27*^AKO^ as treated in (a). Scale bar, 50 μm. (c and d) Gene expression levels of *Fsp27* (c) and *Ccl2* (d) determined by qPCR in 3T3-L1 adipocytes expressing negative control (shNC) or *Fsp27* shRNA (*shFsp27*). (e) Secreted CCL2 in the supernatant from shNC and *shFSP27* 3T3-L1 adipocytes after 24 h culture (*n* = 4). (f) RAW264.7 cells were allowed to migrate towards the conditioned medium from shNC and *shFsp27* 3T3-L1 adipocytes for 24 h, followed by staining with crystal violet. Three fields from each chamber were counted and averaged. Scale bar, 50 μm (*n* = 4). (g) Representative images of F4/80^+^ (red) stained macrophages of PVAT sections from WT and *Fsp27*^−/−^ mice fed with an HFD. F4/80^+^ (red) stained macrophages are denoted by arrowheads. Scale bar, 50 μm. Quantification of F4/80^+^ macrophages per field is shown on the right (*n* = 4). (h and i) Gene expression of *Fsp27* (h) and *Ccl2* (i) determined by qPCR in 3T3-L1 adipocytes expressing an empty vector (Vector) or *Fsp27*-overexpression vector (*Fsp27*-OE) (*n* = 3). (j) Secreted CCL2 in the supernatant from 3T3-L1 adipocytes overexpressing an empty vector or *Fsp27* after 24 h culture (*n* = 4). (k) RAW264.7 cells were allowed to migrate towards the conditioned medium from 3T3-L1 adipocytes overexpressing an empty vector or *Fsp27* for 24 h in the presence or absence of RS102895 (5 μmol/L). Three fields from each chamber were counted and averaged (*n* = 4). Scale bar, 50 μm. (l and m) The concentration of CCL2 was determined by ELISA (l) and the capacity of the conditioned medium to stimulate RAW264.7 cell migration was assessed by a transwell assay (m). PVAT was isolated from both WT and *Fsp27*^−/−^ mice that had been fed with an HFD for three months, and conditioned medium from the PVAT was collected. Data were expressed as means ± SEM. *P* values were calculated by Student’s *t*-test (a−m) and two-way ANOVA with Bonferroni test (k). ns, not significant.

We further reasoned that the decrease in macrophage recruitment to the aortas observed in *Fsp27*^AKO^ mice was probably attributed to the reduced secretion of chemokines by perivascular adipocytes. One such chemokine, CCL2 (also known as monocyte chemoattractant protein-1, MCP1), a potent recruiter of monocytes and macrophages, was shown to be downregulated in the aortas of *Fsp27*^AKO^ mice compared to that of *Fsp27*^fl/fl^ mice (Fig. 4m). We confirmed that CCL2 expression was downregulated in the aortas of *Fsp27*^AKO^ mice as determined by qPCR and immunohistochemistry (Fig. 5a and b). This led us to hypothesize that FSP27 plays an important role in regulating *Ccl2* expression in adipocytes. To investigate this potential role of FSP27, we used lentiviral vector-based short hairpin RNA (shRNA) to silence the expression of FSP27 in 3T3-L1 pre-adipocytes and induced adipocyte differentiation. This resulted in efficient suppression of FSP27 expression in differentiated 3T3-L1 adipocytes (Fig. 5c). Knockdown of FSP27 led to a reduction in the expression and secretion of CCL2, as determined by qPCR and enzyme-linked immunosorbent assay (ELISA) (Fig. 5d and e).

To confirm the effects of FSP27 on macrophage recruitment, we collected the conditioned culture medium from scrambled shRNA lentivirus (shNC) and FSP27-targeting lentivirus (shFSP27)-transduced 3T3-L1 adipocytes and performed *in vitro* transwell assays with mouse RAW264.7 cells. We found that the conditioned medium from *shFSP27* adipocytes significantly reduced macrophage infiltration compared to that from shNC 3T3-L1 adipocytes (Fig. 5f). These results led us to investigate the possibility that FSP27 deficiency in adipocytes may result in reduced macrophage infiltration in PVAT. We performed whole-mount staining of PVAT from WT and *Fsp27*^**−/−**^ mice fed with an HFD, and the results showed that PVAT-infiltrated macrophages were significantly reduced in *Fsp27*^**−/−**^ mice compared to that of WT mice (Fig. 5g). Furthermore, overexpression of FSP27 in 3T3-L1 adipocytes increased the expression and secretion of CCL2 (Fig. 5h−j), leading to an increase in macrophage infiltration compared to the controls (Fig. 5k). To confirm that this effect is mediated by the CCL2/C-C motif chemokine receptor 2 (CCR2) axis, we used a CCR2-selective antagonist RS102895 in the transwell assay. We found that blockade of CCR2 signaling in RAW264.7 cells significantly decreased macrophage migration when cultured with the supernatant from FSP27-overexpressing 3T3-L1 adipocytes (Fig. 5k).

To further assess the direct impact of FSP27 deficiency on CCL2 production and macrophage migration in PVAT, we collected PVAT from WT and *Fsp27*^**−/−**^ mice subjected to an HFD for two months, and then collected conditioned medium from these PVAT samples and determined the levels of CCL2. The results revealed a significant reduction in CCL2 levels in the conditioned medium derived from *Fsp27*^**−/−**^ PVAT (Fig. 5l). We next analyzed the capacity of the conditioned medium to stimulate macrophage migration using a transwell assay. The results showed that the number of migrated macrophages cultured with conditioned medium from *Fsp27*^**−/−**^ PVAT was dramatically decreased (Fig. 5m). Subsequently, the capacity of PVAT to induce macrophage migration was assessed using the transwell co-culture system, in which RAW264.7 cells were incubated with PVAT from WT and *Fsp27*^**−/−**^ mice fed with an HFD. The results showed that the number of migrated cells in *Fsp27*^**−/−**^ group was much reduced than that of WT group (Supplementary Fig. S7). These results suggest that FSP27 deficiency can negatively impact the ability of PVAT to induce macrophage recruitment. In addition, vascular smooth muscle cells (VSMCs) can also adopt a pro-inflammatory phenotype and secrete inflammatory factors including CCL2. To determine whether FSP27 deficiency in adipocytes affect CCL2 production from VSMCs, we isolated PVAT from WT and *Fsp27*^**−/−**^ mice that were fed with an HFD for two months, and then cultured primary VSMCs with the conditioned medium collected from WT and *Fsp27*^**−/−**^ PVAT samples. CCL2 expression and secretion were shown to be comparable between VSMCs treated with conditioned medium from WT and *Fsp27*^**−/−**^ PVAT samples (Supplementary Fig. S8). Moreover, to understand the clinical relevance of CIDEC/CCL2 axis in AAA progression, we analyzed the expression levels of *CIDEC/FSP27* and *CCL2* in a published human AAA study [28]. Microarray data from 59 samples including 10 control organ donors, 20 patients with small AAAs, and 29 patients with large AAAs were obtained. The levels of both *CIDEC *and *CCL2* were significantly upregulated in large AAA sections compared to control aortas (Supplementary Fig. S9). Together, these data indicate that FSP27 promotes the expression and secretion of CCL2 by adipocytes, leading to an increase in macrophage migration through the CCL2/CCR2 axis.

### FSP27 enhances CCL2 expression through c-Jun N-terminal kinase (JNK) activation

**Figure 6:**
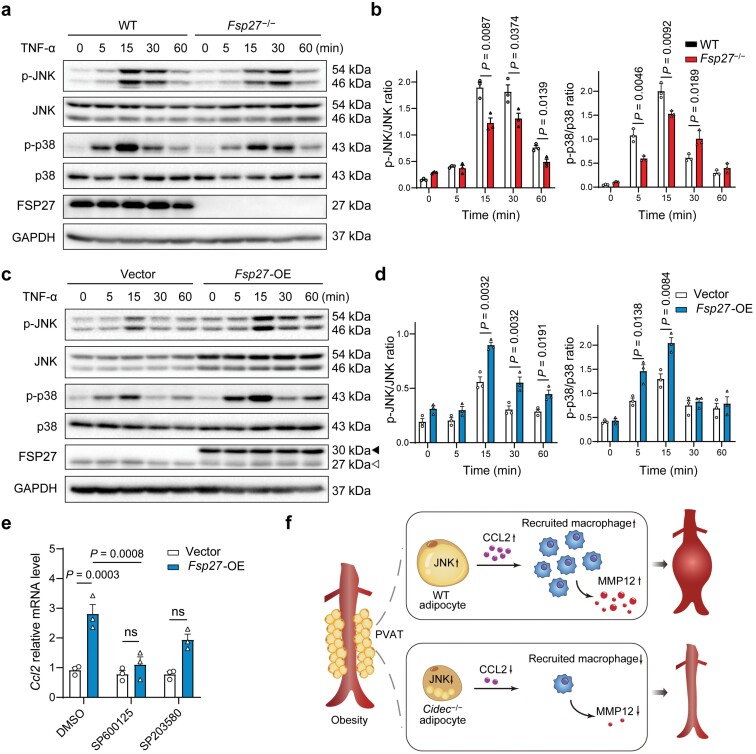
FSP27 enhances CCL2 expression via JNK pathway. (a) WT and *Fsp27*^−/−^ primary adipocytes (adipose-derived SVF) stimulated with TNF-α for the indicated times. Cell lysates were analyzed for phosphorylation levels of JNK and p38 by western blot analysis. (b) Corresponding densitometry analyses of (a) (*n* = 3). (c) Differentiated control and *Fsp27*-overexpressing 3T3-L1 cells were treated with TNF-α and analyzed for phosphorylation levels of JNK and p38 by western blot analysis. Exogenous and endogenous FSP27 were indicated by black arrowhead and white arrowhead, respectively. (d) Corresponding densitometry analyses of (c) (*n* = 3). (e) The expression of *Ccl2* determined by qPCR (*n* = 3). 3T3-L1 adipocytes overexpressing an empty vector (Vector) or *Fsp27* (*Fsp27*-OE) were treated with SP600125 (20 μmol/L) or SP230580 (20 μmol/L) for 24 h. (f) Schematic model showing that CIDEC/FSP27 promotes obesity-related AAA by modulating adipocyte-derived CCL2 secretion and macrophage infiltration through JNK signaling. Data are expressed as means ± SEM. *P* values were calculated by Student’s *t*-test (b and d) and two-way ANOVA with Bonferroni test (e and f). ns, not significant.

The activation of mitogen-activated protein kinases (MAPKs), including JNK and p38, was suggested to mediate CCL2 expression and secretion in many cell types including adipocytes [29–31]. We thus investigated whether JNK and p38 signaling are regulated by FSP27 in adipocytes. In primary adipocytes isolated from *Fsp27*^**−/−**^ mice, TNF-α stimulated JNK and p38 phosphorylation was significantly decreased compared to that from WT mice (Fig. 6a and b). Conversely, overexpression of Fsp27 in 3T3-L1-derived adipocytes resulted in elevated phosphorylation levels of JNK and p38 (Fig. 6c and d). To address the possible role of specific members of the MAPK family in mediating this process, we tested the process with chemical inhibitors of JNK and p38. We observed that JNK inhibition by SP600125, but not p38 inhibition by SP230580, significantly abrogated Fsp27 overexpression-induced *Ccl2* expression in 3T3-L1 adipocytes (Fig. 6e). These results suggest that CIDEC/FSP27 promotes *Ccl2* expression by activating JNK pathway.

## Discussion

Obesity is an important risk factor for AAA, but the underlying mechanisms remain unclear. The identification of genes that regulate PVAT function in the context of obesity is crucial to understanding the correlation between obesity and AAA. In this study, we describe and dissect the functional importance of CIDEC/FSP27 in mediating PVAT expansion and AAA progression. We demonstrate that genetic deletion of *Fsp27* both in whole body and in adipocytes was sufficient to abrogate HFD/Ang Ⅱ-triggered AAA growth, and CIDEC/FSP27 promotes adipocyte-derived CCL2 production through JNK signaling (Fig. 6f). Modulation of FSP27/CCL2 axis may be a novel therapeutic target for obesity-related AAA.

Obesity was reported to increase the incidence of AAA. In a Swedish population-based study, a positive correlation was found between waist circumference and the risk of developing AAA [32]. Specifically, in a large US cohort of middle-aged and older men, the multivariable hazard ratio (HR [95% confidence interval (95% CI)]) for newly diagnosed AAA was 1.30 for baseline BMI 25–30 kg/m^2^ and 1.69 for BMI ≥ 30 kg/m^2^ compared to men who had BMI < 25 kg/m^2^ [13]. PVAT, characterized by low-grade inflammation and dysfunction in the context of obesity, has been considered to be a critical link between obesity and AAA [17]. Importantly, transcriptome profiling in patients with AAA has revealed that immune-response genes are strongly overrepresented in PVAT of AAA compared with PVAT of the non-dilated aortas [33–35]. Infiltrated inflammatory cells and expression of proteases were observed in PVAT adjacent to human AAAs [36]. In our study, we have discovered a mechanistic respect of obesity-driven AAA formation that is mediated by CIDEC/FSP27, an LD-associated protein mainly expressed in adipocytes, acting as a pivotal regulator of aortic inflammation and vascular remodeling. We put mice on an HFD to induce obesity and then infused mice with Ang Ⅱ according to previous study [17]. We have discovered that global deletion or adipocyte-specific deficiency of *Fsp27* provides protection against obesity-driven AAA in mice. Transcriptome profiling has revealed that FSP27 deficiency downregulates MMP12 and macrophage infiltration in the aortas. Mechanistically, *in vitro* findings have shown that FSP27 promotes the expression and production of CCL2 by adipocytes via JNK signaling, leading to subsequent macrophage infiltration to PVAT and the aortic walls. These observations reinforce the causal link between PVAT inflammation and AAA progression.

According to the studies of our group and others, in mice fed with HFD, FSP27 deficiency-related defective adipose lipid storage results in increased circulating triglyceride and insulin resistance [24, 37], both of which are established risk factors for AAA. However, our results demonstrated a remarkable decrease in AAA incidence in *Fsp27*^−/−^ and *Fsp27*^AKO^ mice subjected to HFD and Ang Ⅱ treatment compared to the controls. This finding implies that the anti-inflammatory effect of p53 deficiency may mitigate the impact of FSP27 deficiency-related hypertriglyceridemia and insulin resistance on AAA progression.

Obesity is marked by chronic, low-grade inflammation, primarily driven by the infiltration of macrophages into adipose tissues [38, 39]. Inflammatory infiltration is a major pathological feature of AAA in animal and human studies [40–42]. Infiltrated macrophages, found in the media and adventitia of the aorta, have been shown to promote AAA progression through aggravating inflammation and vascular remodeling [42]. The majority of macrophages that accumulate in the aortic wall during AAA progression probably derive from circulating monocytes mobilized in response to chemokines such as CCL2 [41, 42]. The expression of CCL2 in aneurysmal tissue is significantly elevated in both mouse models and patients with AAA, suggesting its importance in AAA progression [43]. Mice lacking CCR2 are protected from AAA due to the limited recruitment of monocytes to the aorta [44]. Small interfering RNA (siRNA)-mediated inhibition of CCR2, the receptor of CCL2, leads to reduced AAA formation and macrophage infiltration [45]. Pro-inflammatory chemokines and cytokines derived from PVAT in the aortic wall might be important sources contributing to the development of AAA [46]. For example, the expression levels of CCL2 were found to be dramatically increased in PVAT of HFD-fed mice compared to chow diet-fed mice [17]. CCL2 is secreted by various cell types, including endothelial cells, smooth muscle cells, leukocytes, and adipocytes [47]. Our study found that CCL2 was significantly reduced in PVAT from *Fsp27*^AKO^ mice, leading to reduced macrophage infiltration and MMP12 production. In *in vitro* experiments, FSP27 overexpression in 3T3-L1 adipocytes induced CCL2 expression and secretion, while knockdown of *Cidec* resulted in reduced CCL2 expression and secretion. Thus, adipocytes and macrophages appear to work collaboratively to form a positive feedback loop to promote AAA progression.

MMPs have been implicated in the development and progression of aortic disease like AAA and aortic dissections [48, 49]. Specifically, MMP2 and MMP9 have been implicated as key players in aneurysmal expansion [50]. However, we showed that when mice were fed with an HFD and infused with Ang Ⅱ, the expression of *Mmp9* was barely detectable, suggesting that MMP9 is probably not involved in obesity-related AAA progression. Instead, we found that *Mmp12* was specifically increased in mice fed with an HFD. MMP12 is prominently expressed by aneurysm-infiltrating macrophages within the degenerating aortic media of patients with AAA [27, 51], as well as in the aortas from mice treated with CaCl_2_ or treated with Ang Ⅱ and anti-transforming growth factor-β (TGF-β) antibody [26, 52]. In CaCl_2_-induced murine aneurysm model, *Mmp12* deficiency significantly attenuates aneurysm growth [18]. Notably, a recent study suggested that *Mmp12* deficiency prevents vascular remodeling and AAA rupture in *Apoe*^**−/−**^ mice infused with Ang Ⅱ [53]. We further found that *Mmp12* was one of the most significantly reduced genes in the aortas of *Fsp27*^**−/−**^ mice. The reduced *Mmp12* expression in *Fsp27*^AKO^ mice might be, at least in part, due to reduced macrophage infiltration to the aortas, although the exact mechanism of how FSP27 in adipocytes signals to upregulate MMP12 production remains to be further established.

The primary effective treatments for AAAs are open surgery and endovascular repair, but these methods may not provide clear benefits for small AAAs. Various therapeutic and preventive drugs have been tested in animals and clinical trials for AAAs, such as statins, β-adrenoceptor antagonists, renin-angiotensin system inhibitors, and doxycycline [54, 55]. However, at present, there is no established drug therapy that can efficiently inhibit the progression of AAAs or reverse small AAAs. Considering the potential roles of PVAT in AAA progression, PVAT is being explored as a potential target for treating AAAs. Notably, a peroxisome proliferator-activated receptor γ (PPARγ) agonist has been shown to reduce MMP12 levels and the inflammatory status of PVAT, leading to significant attenuation in arterial stiffening in a murine study [56]. In our study, we utilized an HFD and Ang Ⅱ-induced mouse AAA model. Although this model has not been widely used in AAA studies yet, its pathological changes are considered to be relevant to the pathophysiological changes in human obesity-related AAA. We showed that depletion of FSP27 in adipose tissue significantly alleviated AAA progression, as evidenced by the inhibition of aortic dilation and remodeling in the infrarenal aorta, thereby establishing a basis for its potential clinical application in AAA treatment. Indeed, targeting adipose progenitor cells through gene therapy shows promise for clinical feasibility. For instance, delivery of human *BSCL2* gene via adeno-associated virus in a pre-clinical mouse model of congenital generalized lipodystrophy has shown promising results [57]. Further studies are needed to assess the clinical efficacy of FSP27 depletion in AAA treatment.

One limitation of our study is that we were unable to use PVAT-specific FSP27-deficient mice due to the unavailability of PVAT-specific Cre lines. To assess the direct impact of FSP27 on PVAT, we collected PVAT from both WT and *Fsp27*^**−/−**^ mice subjected to an HFD for two months. The CCL2 levels in the conditioned medium, along with the capacity of this medium from *Fsp27*^**−/−**^ PVAT to induce macrophage migration, were notably decreased. Subsequent co-culture experiments with macrophages also demonstrated a significant reduction in the capacity of *Fsp27*^**−/−**^ PVAT to stimulate macrophage migration. These results suggest that FSP27 deficiency in PVAT alone can negatively impact macrophage recruitment. Furthermore, the occurrence of TAAD was similar in both WT and *Fsp27*^**−/−**^ groups, although FSP27-deficient mice exhibited a non-significant increase in the luminal diameter of the descending aorta. Interestingly, WT PVAT resembled BAT and FSP27-deficient PVAT resembled WAT. The varying susceptibility of the thoracic and abdominal aortas to aneurysms in the context of FSP27 deficiency suggests that the markedly decreased AAA incidence in FSP27-deficient mice may be partly due to changes in the inflammatory status of abdominal PVAT. To further study the role of PVAT in AAA progression independent of whole-body adipose tissue, it would be worthwhile to identify PVAT-specific marker genes and develop PVAT Cre driver lines.

In summary, we demonstrate that loss of CIDEC/FSP27 both in the whole body and in adipose tissue leads to a significant reduction of incidence and dilation of AAA after HFD treatment and Ang Ⅱ infusion. Transcriptome profiling indicates that inflammatory responses are reduced in *Fsp27*^**−/−**^ mice. CIDEC/FSP27 promotes PVAT inflammation by inducing CCL2 expression, which subsequently induces macrophage infiltration and MMP secretion. These results provide new mechanistic insights on PVAT inflammation during AAA progression.

## Materials and methods

### Mice

*Fsp27*-null (*Fsp27*^**−/−**^) mice on the C57BL/6J background were generated by our group [58]. *Fsp27*^fl/fl^ mice were kindly provided by Dr. Gonzalez [37]. *Fsp27*^AKO^ mice were generated by crossing *Fsp27*^fl/fl^ and adiponectin-Cre mice from the Jackson Laboratory. All mice were maintained at a constant temperature of 22°C and 60%−65% humidity with a 12-h dark/12-h light cycle in the pathogen-free animal facility. All experiments were carried out in accordance with the guidelines of Fudan University Animal Care Committee for the use and care of laboratory animals. Our study conformed to the Guide for the Care and Use of Laboratory Animals published by the U.S. National Institutes of Health.

### PVAT isolation

The 10- to 12-week-old male mice were placed on a chow diet or an HFD (Research Diet, RD12492) for three months. Animals were sacrificed with an inhalation overdose (5%) of isoflurane. After opening the abdominal cavity, the abdominal aorta was cut from the diaphragm to the infrarenal branch. Aortic tissues were put under a stereomicroscope and in a Petri dish filled with saline. Periaortic adipose tissue was then carefully separated from the aortas.

### Aneurysm induction

For establishment of obesity-induced AAA model, 10- to 12-week-old male mice were placed on an HFD (Research Diet, RD12492) for three months and then infused with Ang Ⅱ or saline. Briefly, a mini osmotic pump (Alzet, Model 1004, DURECT Corporation, Cupertino, CA) loaded with Ang Ⅱ (A9525; Sigma, St. Louis, MO) or saline was implanted into the subcutaneous space through a small incision in the dorsum of the neck. The injection was performed at a rate of 1 μg/kg/min. During surgery, anesthesia was maintained with inhaled isoflurane (2.5%). All animals received buprenorphine at 0.25 mg/kg before surgery and every 12 h for 48 h after surgery. The suprarenal aortic diameters of mice that survived to the end of Ang Ⅱ infusion were examined by ultrasonography. Mice were initially anesthetized using 3% isoflurane. Ultrasonic B-mode images of the abdominal aortas were obtained in mice anesthetized with 2% isoflurane using a Vevo 2100 Imaging System (Visual Sonics, ON, Canada) equipped with a 40-MHz probe. Long-axis scans of the aortas were performed on the abdominal aortas from the left renal arterial branch level to the suprarenal region. Two-dimensional abdominal images of the abdominal aortas were acquired and measured to determine the maximal diameter in the suprarenal region of the abdominal aortas. Aneurysm formation was identified as an increase in the outer width of the suprarenal aorta by at least 50% or greater compared with that in saline-treated mice. After animals were sacrificed with an inhalation overdose (5%) of isoflurane, aortas were harvested and fixed in 4% paraformaldehyde, and the maximum external aortic diameters were photographed and measured. The tissues were then processed for pathological assessment and other biochemical assays.

### Measurement of systolic blood pressure

Systolic blood pressure was measured in mice using the tail-cuff system (Kent Scientific). Mice were placed in the temperature-controlled restrainer for 15 min. Blood pressure was then measured repeatedly and recorded on a data acquisition system (PowerLab, 16/s, ADInstruments). This measurement was performed one day before pump implantation as baseline and on the 28th day after Ang Ⅱ infusion. Systolic blood pressure was averaged from five consecutive measurements.

### Histology and immunohistochemistry

PVAT and aortic samples isolated from the mice were fixed with 4% paraformaldehyde for 24 h and embedded in paraffin. Serial sections (5 μm each) were created at intervals of approximately 500 μm. Paraffin sections were further used for hematoxylin and eosin (H&E) staining or immunohistochemistry. For immunohistochemistry staining, sections were deparaffinized and rehydrated sequentially in xylene, 100% ethanol, 90% ethanol, 70% ethanol, and distilled water, and then autoclaved in 10 mmol/L sodium citrate buffer (pH 6.0) for antigen retrieval at 121°C for 15 min. Sections were treated with 3% hydrogen peroxide to quench endogenous peroxidase activity at room temperature for 10 min and then blocked with 2% bovine serum albumin (BSA) at room temperature for 1 h. Next, aortic sections were incubated with anti-MMP12 antibody (Bioss), anti-CCL2 antibody (Bioss), and anti-F4/80 antibody (CST) at 4°C overnight, and incubated with secondary antibodies at 37°C for 30 min. Finally, the sections were stained with diaminobenzidine and counterstained with H&E.

### RNA-seq

Total RNA was extracted from aortic samples containing PVAT from *Fsp27*^fl/fl^ and *Fsp27*^AKO^ mice subjected to an HFD and Ang Ⅱ treatment. RNA library was prepared with TruSeq RNA Library Prep Kit v2 (Illumina) and 50 bp non-stranded single-end sequencing was performed on a HiSeq 4000 platform (Illumina), with an average of 43 million reads for each sample. RNA-seq read mapping was performed. Gene expression quantification was performed using Salmon v0.14.0, and differential expression analysis was performed with DESeq2 package in R. The DEGs were identified by fold change values greater than 2 and *P* < 0.05. Genes were mapped to the HALLMARK gene set in the Molecular Signatures Database (MSigDB) for pathway analysis.

### RNA quantification

Total RNA from mouse abdominal aortic tissues or cultured cells was extracted using TRIzol reagent (Invitrogen). Isolated RNA was reverse-transcribed to cDNA using reverse transcriptase (TaKaRa Biotechnology, Dalian, China). qPCR was performed with the SYBR Green PCR system in an ABI Q5 thermal cycler (Applied Biosystems, USA). The relative mRNA expression was normalized to *β-actin* and assessed by the 2-^ΔΔCt^ method. The primer sequences used are listed in supplemental material online (Supplementary Table S1).

### ELISA

The concentration of CCL2 in the supernatant from 3T3-L1 adipocytes was quantified using ELISA kit according to the manufacturer’s protocols. Optical density values were measured at a wavelength of 450 nm in an ELISA plate reader.

### Plasmid construction

Full-length *Fsp27* cDNA construct was generated by our group [21]. For lentiviral overexpression, *Fsp27* cDNA was subcloned into pCDH-EF1-MCS-IRES-Puro (System Biosciences). For knockdown, shRNA of *Fsp27* was designed and cloned into pLKO.1 (Addgene, 10878).

### Primary VSMC isolation

Aortas were collected from WT eight-week old mice. After removing the adipose tissue, the aortas were incubated in Dulbecco’s modified Eagle’s medium (DMEM) with 1 mg/mL collagenase Ⅱ (Sigma) at 37°C for 10 min. Adventitia were removed under a dissecting microscope. The remaining aortas were cut into small pieces and further digested with DMEM containing 1.5 mg/mL collagenase Ⅱ and 0.5 mg/mL elastase (Thermo Fisher Scientific) at 37°C for 1 h, with gentle shaking every 10 min. The isolated cells were then washed and plated in complete medium (DMEM-low glucose containing 20% fetal bovine serum (FBS), 100 IU/mL penicillin (Gibco BRL), and 100 μg/mL streptomycin (Gibco BRL)). Studies were performed using passage 3−5 cells.

### Cell culture and treatment

HEK293T cells, 3T3-L1 preadipocytes, and RAW264.7 cells were cultured in DMEM (Thermo Fisher) supplemented with 10% FBS (Gibco BRL), 100 IU/mL penicillin (Gibco BRL), and 100 μg/mL streptomycin (Gibco BRL) at 37°C in a humidified atmosphere containing 5% CO_2_. Primary stromal vascular fractions (SVFs) and 3T3-L1 pre-adipocytes were cultured and differentiated into mature adipocytes using a standard hormone cocktail as previously described [59].

### *Ex vivo* fat tissue explant culture and co-culture experiments

WT and *Fsp27*^−/−^ mice subjected to HFD were sacrificed, and equal amounts of PVAT were rinsed in phosphate-buffered saline (PBS) and incubated in serum-free DMEM supplemented with 1% penicillin-streptomycin for 48 h. Co-culture experiments were conducted in 12-well transwell plates with 0.8-μm pore-sized filters (Corning Costar, USA). Adipose tissues were placed in the bottom chamber and RAW264.7 cells were seeded to the insert.

### Adipose tissue explant conditioned media

PVAT was isolated from WT and *Fsp27*^−/−^ mice fed with an HFD for three months. PVAT (20 mg) was incubated as an explant in serum-free DMEM supplemented with 1% penicillin-streptomycin, maintained at 37°C in a 5% CO_2_ atmosphere for 24 h. The explant culture media were subsequently collected, centrifuged, and frozen at −80°C as whole adipose tissue explant conditioned media.

### Transwell assay

Transwell assay was assessed using 8-μm transwell filters (Millipore, Billerica, MA, USA) in a 12-well plate. Macrophages were added into the upper chamber containing basic culture medium without serum, and the lower chamber was filled with conditioned culture medium from 3T3-L1 adipocytes. Macrophage migration was determined 24 h later. Cells on the lower surface of the membrane were fixed with 4% paraformaldehyde and stained with 0.1% crystal violet. The number of infiltrating cells was counted in five randomly selected microscopic fields of each filter.

### Western blot analysis

Mouse aortic tissue or cellular proteins were extracted using radioimmunoprecipitation assay (RIPA) buffer (25 mmol/L Tris–HCl pH 7.6, 150 mmol/L NaCl, 1% NP-40, 1% sodium deoxycholate, and 0.1% SDS) containing protease inhibitors (Roche). Protein samples were electrophoresed on 10% SDS-PAGE and transferred onto polyvinylidene fluoride (PVDF) membranes. Then, the membranes were blocked with 5% BSA in TBST and incubated with primary antibodies at 4°C overnight. Membranes were washed with TBST and incubated with a horseradish peroxidase-conjugated secondary antibody. Protein bands were detected by the ECL-plus system (Thermo Fisher).

### Lentivirus production and infection

Lentivirus was produced by co-transfecting a knockdown or overexpression vector with psPAX2 (Addgene, 12260) and pMD2.G (Addgene, 12259) in HEK293T cells, respectively. Lentiviral particles were collected, concentrated at 70,000 *g* for 2 h or directly aliquoted and stored at −80°C until use. For lentivirus infections, 3T3-L1 preadipocytes at 50%−70% confluence were infected with lentivirus with 8−10 μg/mL polybrene. After 24 h infection, cells were selected against 5 μg/mL puromycin for at least 48 h before further use for the described experiments.

### Statistical analysis

No data were considered an outlier, or removed from the analyses. Blinding was used for data analysis. Significant differences between two independent groups were analyzed using unpaired Student’s *t*-test. The aortic incidence between the two groups was compared using Fisher’s exact test. For multicomparisons, one-way ANOVA was used followed by pairwise comparisons with Bonferroni test. The values are presented as the means ± SEM. *P* < 0.05 was considered statistically significant.

## Supplementary Material

loae035_suppl_Supplementary_Material

## Data Availability

All study data are included in the article and/or supplementary information. Materials are available upon request.
