# Supplementary material for: CIDEC/FSP27 exacerbates obesity-related abdominal aortic aneurysm by promoting perivascular adipose tissue inflammation
Source: Life Metab. 2024 Sep 18;4(1):loae035. doi: 10.1093/lifemeta/loae035 (PMC11770823; doi:10.1093/lifemeta/loae035)
Supplement: loae035_suppl_Supplementary_Material [file loae035_suppl_supplementary_material.docx]

**Supplementary Figures**

**
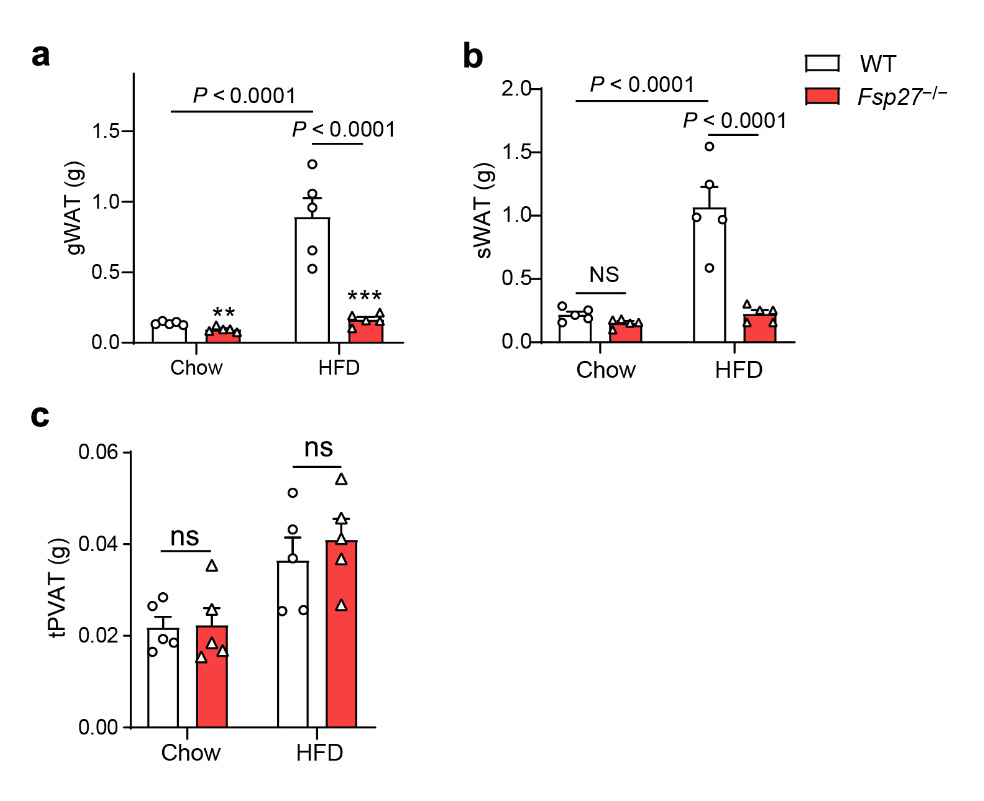
**

**Supplementary Figure S1** Reduced body weight and fat mass of WT and *Fsp27*^−/−^ mice after high-fat diet (HFD) treatment. (a and b) Weight of gonadal WAT (gWAT) (a) and subcutaneous WAT (sWAT) (b) from WT and *Fsp27*^−/−^ mice fed with a chow diet or an HFD for three months. (c) Thoracic perivascular adipose tissue (tPVAT) from mice described in (a) (*n* = 5). Data are presented as the means ± SEM. Significance was established using two-way ANOVA with Bonferroni test. ns, not significant.

**
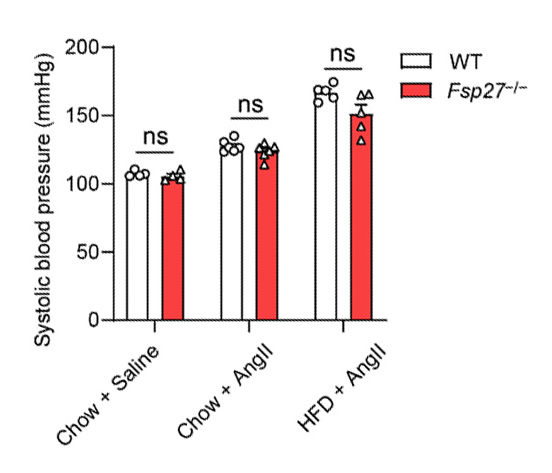
**

**Supplementary Figure S2** Systolic blood pressure of WT and *Fsp27*^−/−^ mice after HFD treatment with or without Ang II infusion. WT and *Fsp27*^−/−^ mice were fed with a chow diet or an HFD for three months and infused with Ang II (1 μg/kg/min). Significance was established using Student’s *t*-test. ns, not significant.


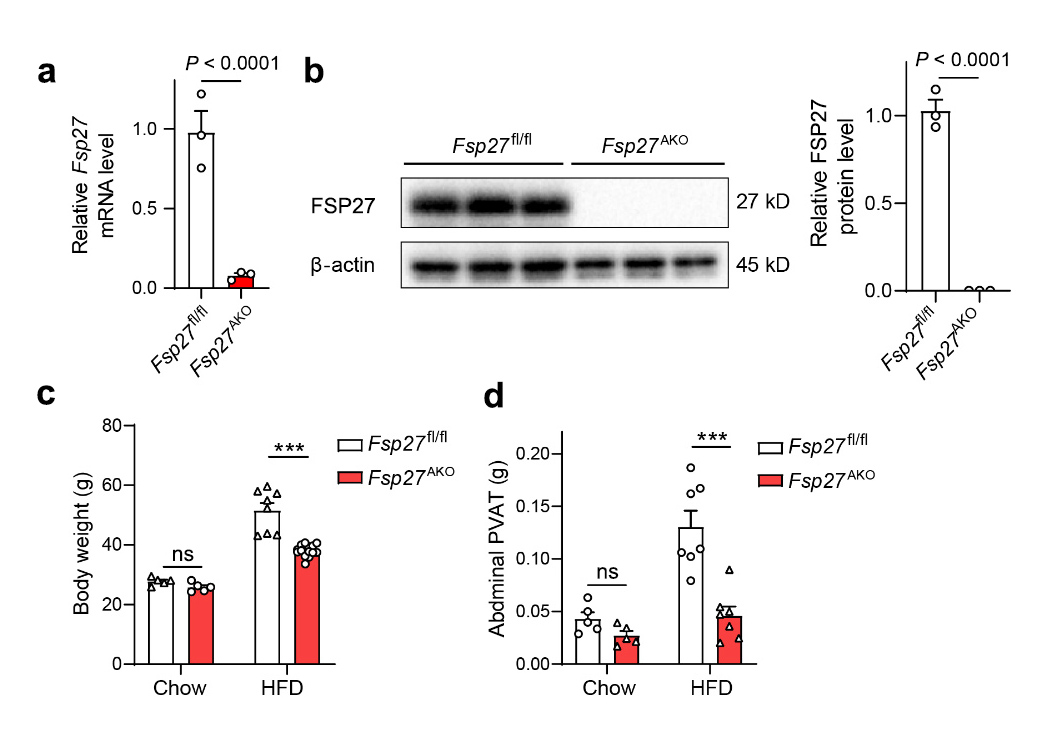


**Supplementary Figure S3** Adipocyte-specific FSP27 deficiency results in reduced body weight and decreased weight of abdominal PVAT. (a) Relative mRNA expression levels of *Fsp27* in abdominal PVAT of *Fsp27*^fl/fl^ and *Fsp27*^AKO^ mice fed with an HFD for four months (*n* = 4 per group). (b) Western blot analysis of FSP27expression in abdominal PVAT from mice as described in (a). Corresponding densitometry analysis is shown on the right. (c) Body weight of *Fsp27*^fl/fl^ and *Fsp27*^AKO^ mice as described in (a). (d) Weight of abdominal PVAT from *Fsp27*^fl/fl^ and *Fsp27*^AKO^ mice as described in (a). Data are presented as the means ± SEM. Significance was established using Student’s *t*-test. ^*^*P* < 0.05, ^***^*P* < 0.001. ns, not significant.


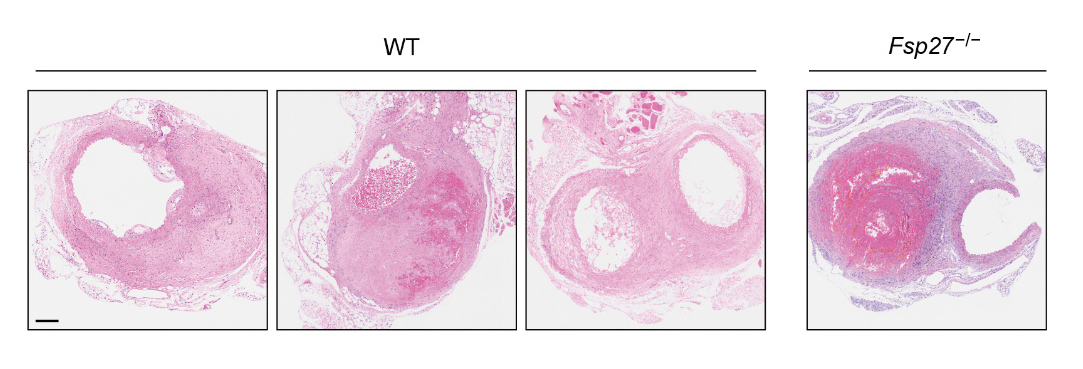


**Supplementary Figure S4** Histology of formed aneurysms from WT and *Fsp27*^−/−^ mice. WT and *Fsp27*^−/−^ mice fed with a HFD for three months were administered with angiotensin II (Ang II) for four weeks. Hematoxylin and eosin (H&E) staining of the formed aneurysms from WT and *Fsp27*^−/−^ mice were shown. Scale bar, 200 μm.


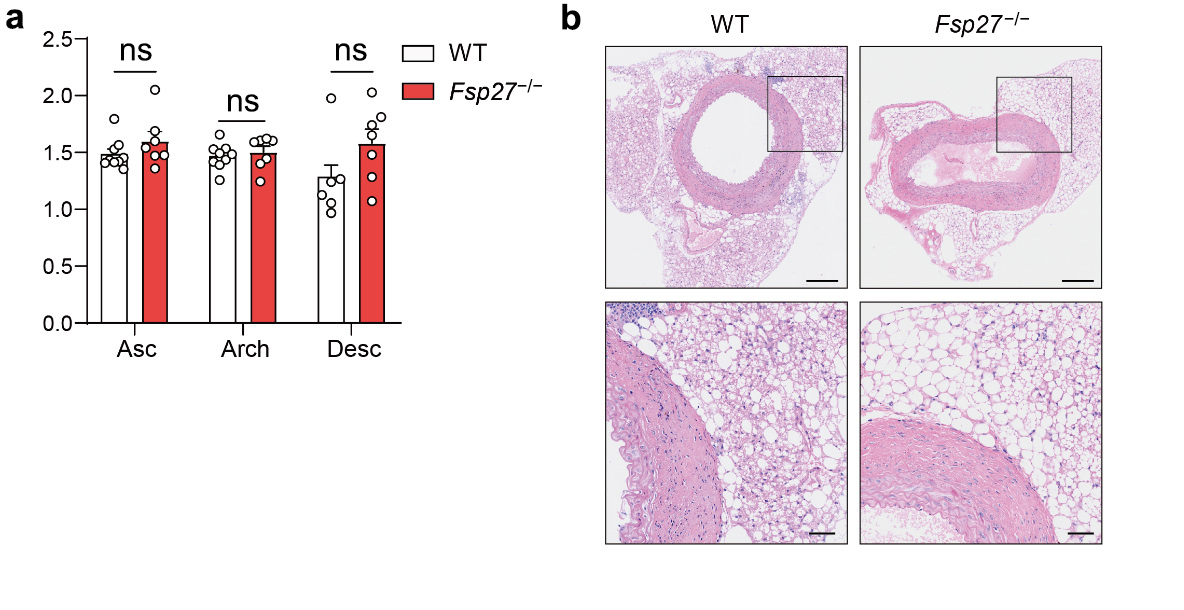


**Supplementary Figure S5** The effect of FSP27 on thoracic aortic aneurysms and dissection (TAAD). WT and *Fsp27*^−/−^ mice fed a chow diet or an HFD for 3 months were administered with Ang II for four weeks. (a) The luminal diameters of the thoracic aortas in WT and *Fsp27*^−/−^ mice were echocardiographyically measured. Asc, ascending; Desc, descending thoracic; Arch, aortic arch. (b) H&E staining of the descending aorta from WT and *Fsp27*^−/−^ mice. Scale bar (Top panel, 200 μm; bottom panel, 50 μm). Data are expressed as means ± SEM. *P* values were calculated by Student’s *t-*test. ns, not significant.


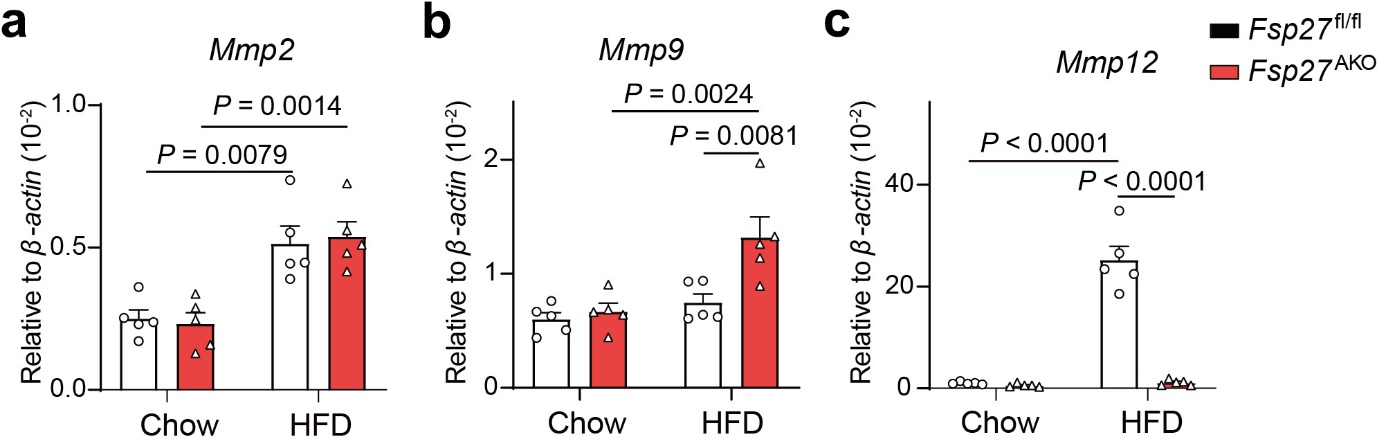


**Supplementary Figure S6** Relative mRNA expression levels of *Mmp2*, *Mmp9*, and *Mmp12* in the aortas of *Fsp27*^AKO^ mice. Relative mRNA expression levels of *Mmp2*, *Mmp9*, and *Mmp12* were determined in the aneurysmal tissues of *Fsp27*^fl/fl^ and *Fsp27*^AKO^ mice fed with a chow diet or a HFD and then infused with or without Ang II (*n* = 5). Data are presented as the means ± SEM. *P* values were calculated by Student’s *t*-test.


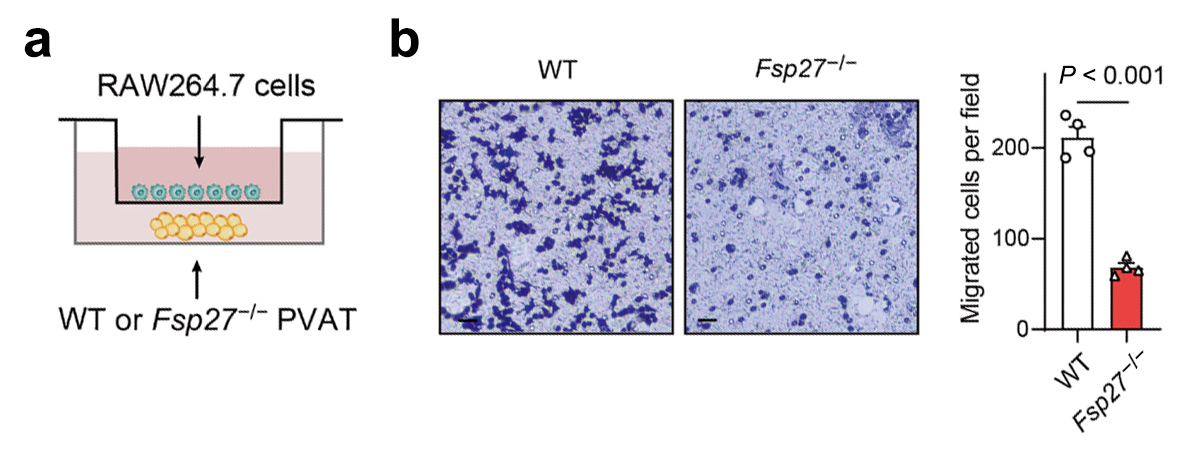


**Supplementary Figure S7 FSP27** deficiency decreases the capacity of PVAT to induce macrophage migration. (a) Schematic illustration of the transwell coculture assay. PVAT is placed in the bottom chamber of the transwell, while RAW264.7 cells are placed in the upper chamber and allowed to migrate towards the PVAT explant, followed by staining with crystal violet. (b) The capacity of RAW264.7 cell migration assessed by a transwell assay. Three fields from each chamber were counted and averaged. Scale bar, 50 μm (*n* = 4). Data are expressed as means ± SEM. *P* values were calculated by Student’s *t*-test.

**
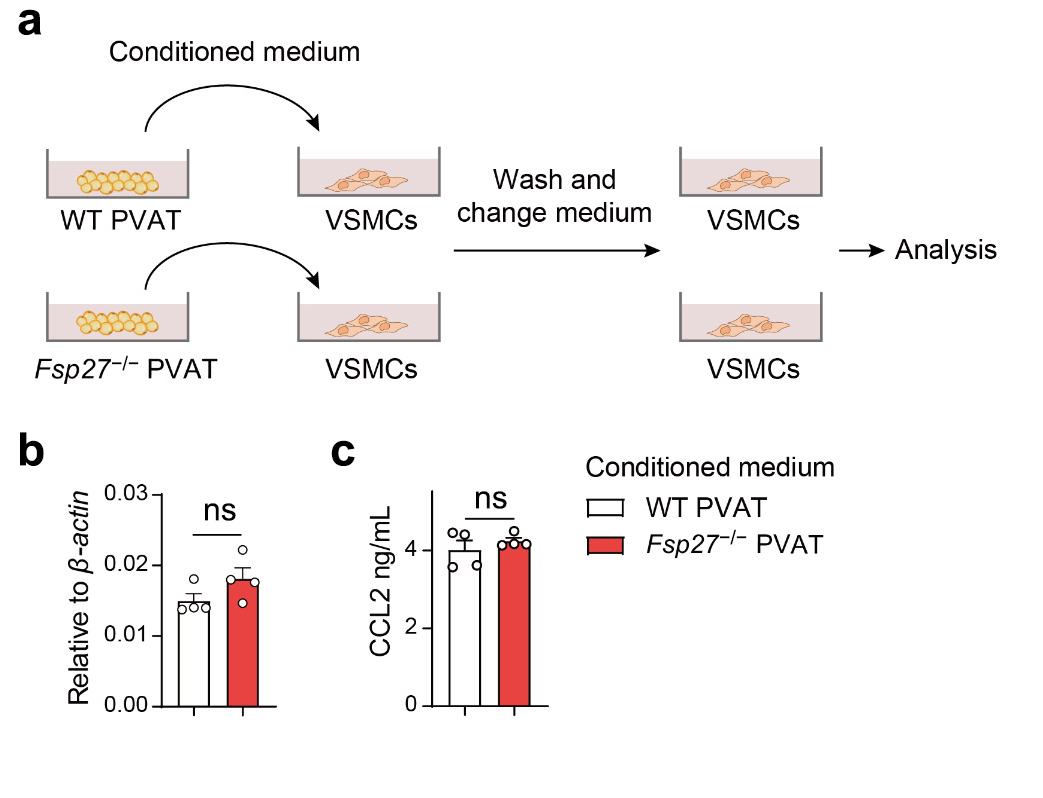
**

**Supplementary Figure S8** The effect of WT or *Fsp27***^−/−^** PVAT on CCL2 secretion from vascular smooth muscle cells (VSMCs). (a) Schematic illustration of experimental design. Primary mouse VSMCs were treated with conditioned medium collected from WT or *Fsp27***^−/−^** PVAT. (b) Relative mRNA expression level of *Ccl2* in VSMCs was determined by quantitative real-time PCR (qPCR) . (c) Levels of CCL2 in the supernatant collected from PVAT-derived conditioned medium treated VSMCs were determined by enzyme-linked immunosorbent assay (ELISA). *P* values were calculated by two-tailed unpaired *t*-test. ns, not significant.


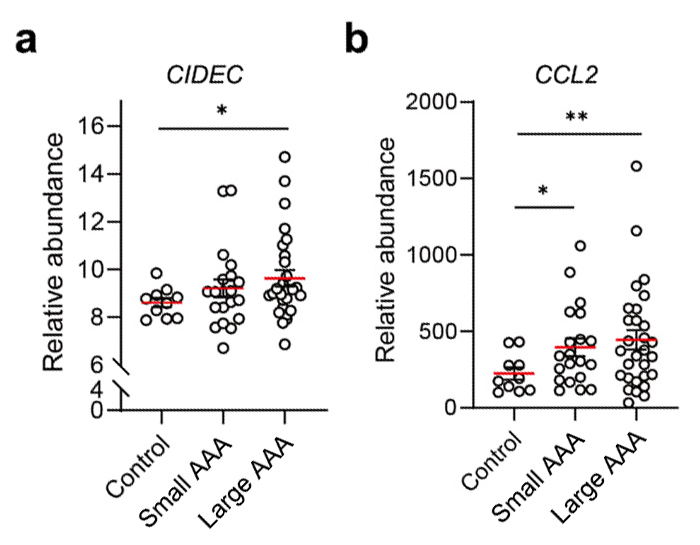


**Supplementary Figure S9** Upregulated *CIDEC* and *CCL2* in human AAAs. Gene expression data including 10 control organ donors (Control), 20 patients with small AAAs (small AAA), and 29 patients with large AAAs (large AAA) were obtained from a public study (GSE57961). Normalized abundances of *CIDEC* and *CCL2* with means are displayed (red). *P* values were calculated by Two-tailed tunpaired *t*-test. ^*^*P* < 0.05; ^**^*P* < 0.01.

**Supplementary Table S1** Primer sequences of qPCR.

| Primer name | Sequence (5' to 3') |
| --- | --- |
| *β-actin*_Forward | ACACTGTGCCCATCTACGAG |
| *β-actin*_Reverse | CAGCACTGTGTTGGCATAGAG |
| *Fsp27*_Forward | GTGTCCACTTGTGCCGTCTT |
| *Fsp27*_ Reverse | CTCGCTTGGTTGTCTTGATT |
| *Ccl2*_Forward | GCCTGCTGTTCACAGTTGC |
| *Ccl2*_ Reverse | GAGTGGGGCGTTAACTGCAT |
| *Mmp2*_Forward | AACGGTCGGGAATACAGCAG |
| *Mmp2*_Reverse | GTAAACAAGGCTTCATGGGGG |
| *Mmp9*_Forward | CCAGCCGACTTTTGTGGTCT |
| *Mmp9*_Reverse | CTTCTCTCCCATCATCTGGGC |
| *Mmp*12_Forward | GCTGTCACAACAGTGGGAGA |
| *Mmp*12_Reverse | TACCAGATGGGATGCTTGGC |
| *Il1b*_Forward | GCAACTGTTCCTGAACTCAACT |
| *Il1b*_Reverse | ATCTTTTGGGGTCCGTCAACT |
| *Il6*_Forward | CCAGAGATACAAAGAAATGATGG |
| *Il6*_Reverse | ACTCCAGAAGACCAGAGGAAAT |
| *F4/80_*Forward | CTTTGGCTATGGGCTTCCAGTC |
| *F4/80*_Reverse | GCAAGGAGGACAGAGTTTATCGTG |
| *Tnfa*_Forward | CCAGACCCTCACACTCAGATC |
| *Tnfa*_Reverse | CACTTGGTGGTTTGCTACGAC |
|  |  |
